# Revision Interposition Arthroplasty with AlloDerm for Persistent Thumb Carpometacarpal Pain Following TightRope Suspensionplasty: A Case Report

**DOI:** 10.1177/22925503261479629

**Published:** 2026-08-26

**Authors:** Brady Duiker, Alexander Platt, Colborne J. Kemna, Brett Ponich, Aaron Knox

**Affiliations:** 1 Cumming School of Medicine, 70401 University of Calgary, Calgary, AB, Canada; 2 Division of Plastic and Reconstructive Surgery, 2129 University of Calgary, Calgary, AB, Canada; 3 Department of Surgery, 70401 University of Calgary, Calgary, AB, Canada

**Keywords:** thumb carpometacarpal arthritis, TightRope suspensionplasty, AlloDerm interposition arthroplasty, revision hand surgery, load-dependent thumb pain, occupational hand injury, case report, rizarthrose, suspensionplastie par implant TightRope, interposition par greffe AlloDerm, chirurgie de révision de la main, douleur du pouce liée à la mise en charge, blessure professionnelle de la main, rapport de cas.

## Abstract

Thumb carpometacarpal (CMC) arthritis causes thumb pain and disability, often requiring surgery when conservative treatments fail. Trapeziectomy with ligament reconstruction and tendon interposition remains a standard approach, but TightRope suspensionplasty offers a tendon-sparing alternative that allows early mobilization. This report discusses a 48-year-old police officer with advanced thumb CMC arthritis who underwent trapeziectomy with TightRope suspensionplasty. Although initially successful, she experienced ongoing pain during high-impact activities, especially when operating a firearm, which is essential for her job. The pain, occurring during thumb adduction and forceful loading, did not improve with conservative treatment. Revision surgery with AlloDerm and hardware removal was performed. Serial follow-up at 3, 6, and 12 months demonstrated progressive strength recovery and stable metacarpophalangeal alignment, with return to occupational duties with ergonomic modifications. This case highlights the potential of biologic interposition to manage load-related pain after suspensionplasty in high-demand individuals.

## Résumé

La rizarthrose, qui est responsable de douleurs et d’une incapacité, doit souvent être opérée après l’échec des traitements conservateurs. La trapézectomie avec reconstruction ligamentaire et interposition tendineuse demeure standard, mais la suspensioplastie par implant TightRope épargne les tendons et favorise une mobilisation rapide. Le présent rapport décrit le cas d’une policière de 48 ans ayant une rizarthrose avancée qui a subi une trapézectomie avec suspensioplastie par implant TightRope. Bien que l’intervention ait d’abord été considérée comme réussie, la patiente éprouvait des douleurs persistantes lors d’activités à fort impact, notamment le maniement d’armes à feu, essentiel à son travail. La douleur, ressentie pendant l’adduction du pouce et une mise en charge énergique, n’a pas diminué malgré un traitement conservateur. Une chirurgie de révision par greffe AlloDerm et retrait de l’implant a été réalisée. Le suivi récurrent au bout de trois, six et 12 mois a démontré une récupération progressive de la force et un alignement métacarpophalangien stable, de même que la reprise des fonctions professionnelles après des modifications ergonomiques. Ce cas démontre le potentiel de l’interposition biologique pour gérer la douleur liée à la mise en charge après une suspensioplastie chez les personnes aux besoins fonctionnels élevés.

## Introduction

Thumb carpometacarpal (CMC) arthritis is the second most common form of hand osteoarthritis.^1^ Laxity of the anterior oblique ligament causes instability, abnormal loading, and cartilage wear, leading to pain, weakness, and reduced function.^1^

Nonoperative management may relieve symptoms but does not alter disease progression.^2^ Among surgical options, trapeziectomy with or without ligament reconstruction and tendon interposition (LRTI) remains the most reliable procedure ^3^ but requires prolonged immobilization.

TightRope suspensionplasty uses a suture-button construct to link the first and second metacarpals, enabling early mobilization without tendon harvest. Clinical outcomes are comparable to LRTI, with shorter operative time and earlier return to activity. As TightRope suspensionplasty is increasingly adopted, persistent post-procedural pain is an emerging problem for which revision strategies remain under-reported.

## Case Presentation

A 48-year-old female police officer presented with longstanding left wrist and thumb pain. Her history included prior ulnar shortening osteotomy, triangular fibrocartilage complex debridement with wrist denervation, and multiple corticosteroid injections, which provided only temporary relief.

In August 2024, the patient underwent left trapeziectomy with TightRope suspensionplasty, removal of ulnar hardware, and distal radioulnar joint ligament reconstruction. Four months postoperatively, she experienced persistent activity-dependent pain during forceful gripping and pinching that impaired essential occupational duties, including firearm discharge. Examination revealed scar thickening and tenderness over the button site with no mechanical instability or metacarpal subsidence on radiograph.

Pain was localized to the area between the thumb and index metacarpal. A targeted local anesthetic injection in this region alleviated the pain, confirming a peripheral soft-tissue source. This was consistent with underlying arthrosis at the metacarpal bases, suggesting that surgical revision with soft-tissue interposition could be beneficial.^4^

In May 2025, she underwent revision surgery to remove the TightRope device and perform AlloDerm interposition arthroplasty. Using the previous surgical incisions, the extensor pollicis brevis (EPB) tendon and joint capsule were incised, and the suture-button construct of the TightRope was removed. The base of the first metacarpal was then circumferentially dissected and cleared of scar tissue. An AlloDerm sheet was placed between the bases of the first and second metacarpals to prevent metacarpal contact during loading. The AlloDerm was then wrapped around the metacarpal base and secured with interrupted 3-0 PDS sutures. Fluoroscopy confirmed proper positioning of the metacarpal with maintained space between the bases of the first and second metacarpals. The EPB tendon and capsule were then re-approximated. The incision was closed in layers, and she was discharged the same day without complications.

Rehabilitation included thumb spica splinting with the interphalangeal joints free and progressive range-of-motion and strengthening exercises. After 1 month, the patient reported significant pain reduction. By 3 months, she had resumed all occupational duties, including firearms training and physical fitness testing. She noted a subjective increase in grip strength. Her upper extremity functional index-15 score,^5^ a validated measure of upper extremity function, improved from 34/100 preoperatively to 75/100 at 3 months, while her grip strength improved to 24 kg (contralateral: 32 kg) and key pinch to 3.6 kg (contralateral: 7.2 kg). At 6 months, grip was 22.4 kg (contralateral 33.1 kg) and key pinch 3.8 kg (contralateral 6.8 kg); MCP demonstrated 30° of hyperextension. At 12 months, grip improved to 26.0 kg (contralateral: 33.3 kg), key pinch to 5.4 kg (contralateral: 9.6 kg), and tripod pinch to 5.0 kg (contralateral: 8.9 kg), with Kapandji opposition 7/10. MCP hyperextension decreased to 21°, and the UEFI at 12 months was 79.3/100. The patient reported at least 96% improvement in pain, with residual discomfort limited to high-volume firearm training (tolerable for at least 150 rounds) and strenuous exercise. Video documentation of firearm operation before and after revision confirmed successful return to occupational proficiency (Supplemental Video 1). Residual limitations included mild dorsal thumb numbness and mild metacarpophalangeal hyperextension, both of which improved over the follow-up period.

## Discussion

TightRope suspensionplasty has demonstrated outcomes equivalent to LRTI, with the advantage of shorter operative times and earlier mobilization.^6–9^ However, device-related pain and persistent mechanical symptoms may necessitate revision.^4^ Known complications include suture impingement, synovitis, and technical challenges of revision once hardware is incorporated in scar tissue. Revision options range from hardware removal alone for localized impingement without joint pathology, to biologic interposition when articular pain is confirmed, to formal ligament reconstruction when instability or metacarpophalangeal deformity is present.^10^

**Figure 1.**
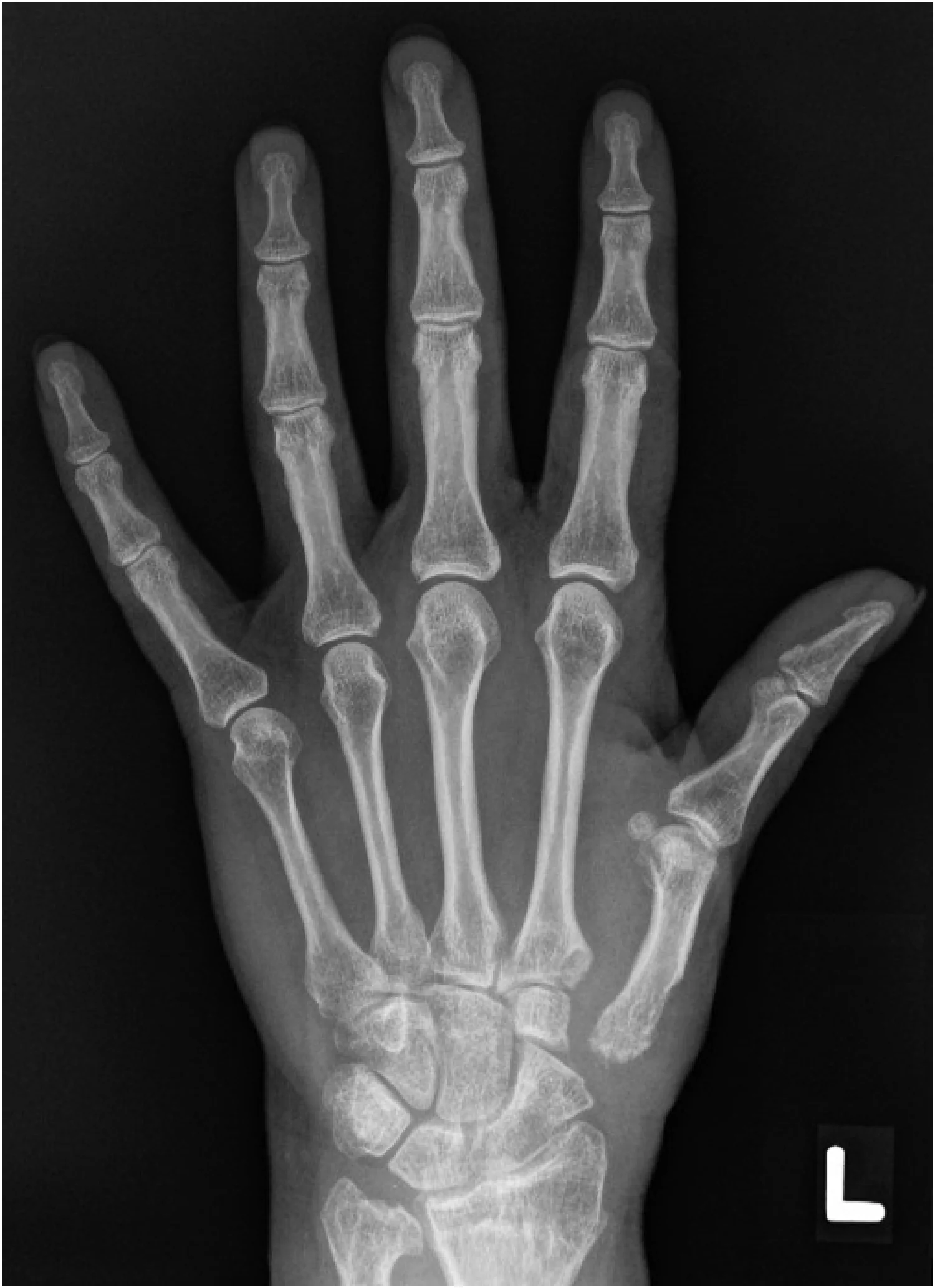
Anteroposterior radiograph of the left hand at 12 months following revision trapeziectomy and AlloDerm interposition arthroplasty. Proximal migration of the first metacarpal is present, which has remained stable compared to the immediate postoperative period and has not correlated with functional deterioration. The trapezoid is intact. The interposition space is maintained with no evidence of metacarpal base impingement.

Unlike typical suspensionplasty failures, linked to pain, subsidence, or instability,^4^ this patient demonstrated activity-dependent symptoms despite preserved radiographic alignment. This presentation suggests a localized mechanical pain generator activated only during forceful loading. Radiographic assessment at 12 months (Figure 1) confirmed stable first metacarpal subsidence, a near-universal finding following trapeziectomy-based arthroplasty not correlated with functional outcomes.^4^

The absence of resting pain and normal thumb stability suggests contact between the first and second metacarpal bases rather than ligament weakness. Interposition arthroplasty served as a spacer, reducing compressive forces and load-related pain.

AlloDerm interposition has previously been described in primary thumb CMC arthroplasty ^4,11^ but not as a revision strategy for persistent, load-dependent pain following TightRope suspensionplasty. This case expands the potential role of biologic interposition beyond primary reconstruction, illustrating its utility as a salvage option for symptomatic metacarpal contact rather than instability or construct failure. MCP hyperextension improved from 30° at 6 months to 21° at 12 months with no adduction deformity, supporting construct stability without formal ligament reconstruction. No additional stabilization was required: scar was cleared only to seat the graft, while the post-trapeziectomy space had consolidated with mature soft tissue that maintained first metacarpal position.

When radiographic stability remains and symptoms depend on activity, a diagnostic local anesthetic block can help identify a mechanical pain source.

## Conclusion

This case shows that AlloDerm interposition is a viable salvage option for high-demand patients with persistent, load-dependent pain after trapeziectomy with TightRope suspensionplasty. Serial assessment at 3, 6, and 12 months demonstrated sustained pain relief, progressive strength recovery, and stable metacarpophalangeal alignment, enabling return to active occupational duties. Biologic interposition is a valuable addition in managing complex revision cases of thumb CMC arthritis. Further prospective studies are needed to evaluate the long-term durability of AlloDerm interposition in revision CMC procedures.

## Supplemental Material

**Figure:** Video 1.
